# Synthesis of Biscoumarin and Dihydropyran Derivatives and Evaluation of Their Antibacterial Activity

**DOI:** 10.3390/molecules200917469

**Published:** 2015-09-18

**Authors:** Jing Li, Chang-Wei Lv, Xiao-Jun Li, Di Qu, Zheng Hou, Min Jia, Xiao-Xing Luo, Xia Li, Ming-Kai Li

**Affiliations:** 1 School of Chemical Engineering, Xi’an University, Xi’an 710065, China; E-Mails: lijing3157@aliyun.com (J.L.); xjli@xawl.edu.cn (X.-J.L.); 2 Department of Orthopaedics, Xijing Hospital, the Fourth Military Medical University, Xi’an 710032, China; E-Mail: lvcwei@fmmu.edu.cn; 3 Department of Pharmacology, School of Pharmacy, the Fourth Military Medical University, Xi’an 710032, China; E-Mails: qu_di1990@126.com (D.Q); hzh_0001@163.com (Z.H.); yangziyi@fmmu.edu.cn (M.J.); xxluo3@fmmu.edu.cn (X.-X.L.); 4 Department of Neurosurgery, Xijing Hospital, the Fourth Military Medical University, Xi’an 710032, China

**Keywords:** biscoumarins, dihydropyran, *S. aureus*, *S. epidermidis*

## Abstract

In an attempt to find a new class antibacterial agents, a series of biscoumarins (**1**–**4**) and dihydropyrans (**5**–**13**) were successfully prepared. The molecular structures of four representative compounds, that is, **4**, **5**, **8** and **12** were confirmed by single crystal X-ray diffraction study. These synthesized compounds were screened for their antibacterial activity *in vitro* against *Staphylococcus aureus* (*S. aureus* ATCC 29213), methicillin-resistant *S. aureus* (MRSA XJ 75302), vancomycin-intermediate *S. aureus* (Mu50 ATCC 700699), USA 300 (Los Angeles County clone, LAC), *Staphylococcus epidermidis* (*S. epidermidis* ATCC 14990), methicillin-resistant *S. epidermidis* (MRSE XJ 75284) and *Escherichia coli* (*E. coli* ATCC 25922). Additionally, there are two classical intramolecular O–H···O hydrogen bonds (HBs) in biscoumarins **1**–**4** and the corresponding HB energies were further performed with the density functional theory (DFT) [B3LYP/6-31G*] method.

## 1. Introduction

*Staphylococcus aureus* (*S. aureus*) is a common pathogen causing serious hospital infections, community acquired infections and is also the pathogen with the highest drug resistant incidence. There are still no specific and selective antibacterial agents to kill MRSA (Methicillin-resistant *S. aureus*), which leads to a high death rate of patients [1]. Resistance to antibacterial drugs has been relentlessly increasing over the past two decades, along with a noticeable decrease in the development of new drugs for infections [2]. Few candidate drugs that offer benefits over existing drugs are available, and few drugs that will treat infections due to the so-called “ESKAPE” pathogens (*Enterococcus faecium*, *Staphylococcus aureus*, *Klebsiella pneumoniae*, *Acinetobacter baumannii*, *Pseudomonas aeruginosa*, and *Enterobacter* spp.) are being developed [3]. The need for newer, more effective treatment options for multidrug-resistant (MDR) pathogens induced the Infectious Diseases Society of America (IDSA) to launch the 10 × 20 initiative against a range of resistant “ESKAPE” pathogens including MRSA and to develop 10 new effective antimicrobial drugs to treat the infections caused by these pathogens by 2020 [4]. Therefore, it is evident and urgent to seek a new defending strategy against MRSA now.

Bisoumarin and dihydropyran derivatives are widely available in nature. These derivatives exhibit various biological activities, such as anticoagulant, insecticidal, antihelminthic, hypnotic, and antifungal activities, phytoalexin production, and HIV protease inhibition [5,6,7,8]. Moreover, the two kinds of compounds are gaining increasing interest because of their versatile activities through chemical modifications (different substituents on the aromatic ring) [9]. For biscoumarins, the minimum active harmacophore reportedly consists of a coumarin dimer containing an aryl substituent on the central linker methylene. The addition of 4-hydroxy substituents to coumarin rings improved the potency of the compounds [10]. Recognizing the considerable importance of the compounds, the researchers focused on the synthesis of bisoumarin and dihydropyran derivatives [11]. 

**Figure 1:**
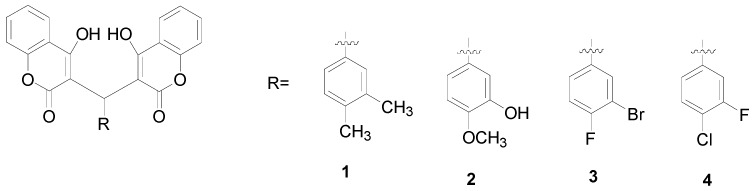

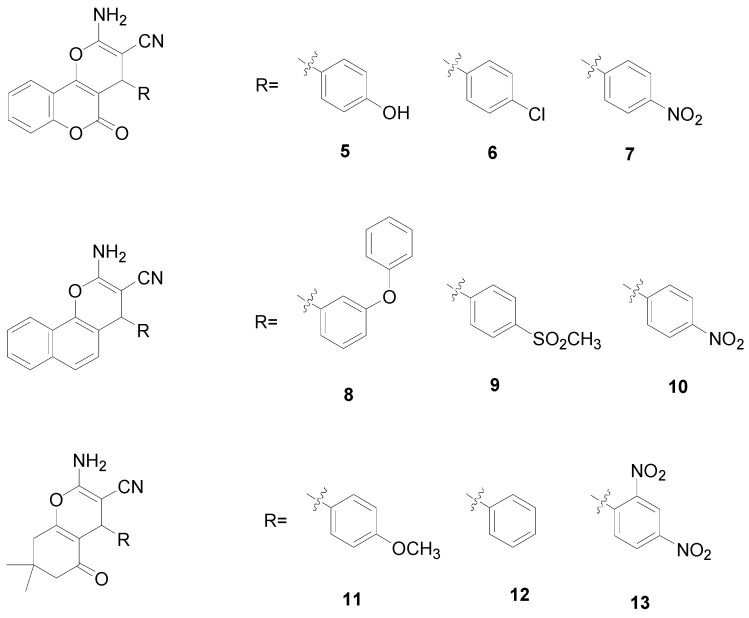
Chemical structures of compounds **1**–**13**.

To identify more active compounds, we prepared a series of biscoumarin and dihydropyran derivatives (Figure 1) and then evaluated their antibacterial activities. Finally, we calculated the total HB energies of compounds **1**–**4** by density functional theory (DFT) method to elucidate a possible relationship between such hydrogen-bonded structures and their antibacterial activities.

## 2. Results and Discussion

### 2.1. Molecular Structure

**Figure 2:**
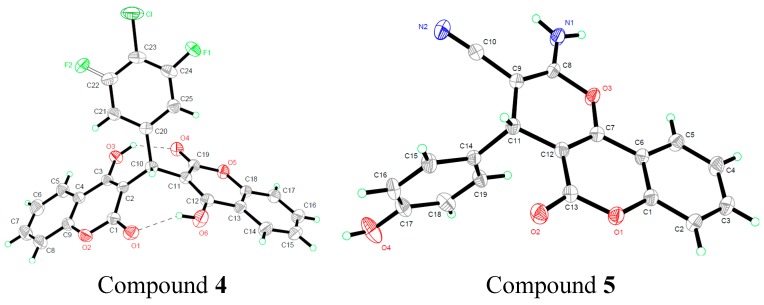

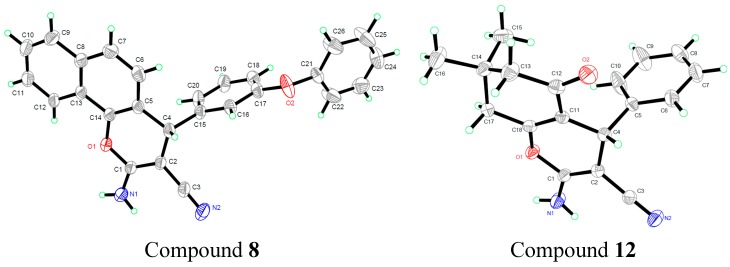
Crystal structure of compounds **4**, **5**, **8** and **12**.

The crystal structures of compounds **4**, **5**, **8** and **12** are given in Figure 2. In the crystal structure of compound **4**, two crystallographically independent molecules are present in the asymmetric unit. The whole molecule is disordered over two orientations with refined site occupancies of 1:1, and two 4-hydroxycoumarin moieties are linked through a methylene bridge on which one hydrogen atom has been replaced with a 3-fluoro-4-chlorophenyl group. However, the two components differ with respect to the reversed twist directions of the 3-fluoro-4-chlorophenyl residue. In the compound, two classical intramolecular hydrogen bonds were found; each links a coumarin hydroxyl and carbonyl group [*d*(O_3_–O_4_) = 2.598 Å, *d*(O_1_–O_6_) = 2.676 Å].

In compounds **5**, **8** and **12**, the new formed pyran ring is essentially planar because the maximum deviation of the atoms in the skeleton from the C_7_-O_3_-C_8_ plane is only 0.2533 Å in compound **5**, the corresponding value is 0.2006 Å and 0.6686 Å in compounds **8** and **12** respectively. The adjacent coumarin ring, naphthalene ring and ketone ring is also basically parallel to the pyran ring in the three compounds. The torsion angle between aromatic ring and pyran ring is about 89.452°, 84.160° and 89.801° in compounds **5**, **8** and **12** respectively.

### 2.2. Quantum Chemical Calculations

#### 2.2.1. Geometric Parameters of Compounds **1**–**4**

**Figure 3:**
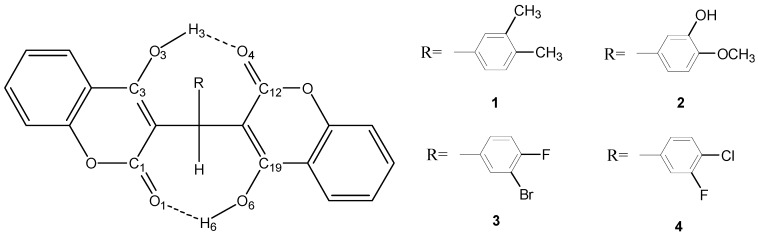
Schematic presentation of compounds **1**–**4**.

The fully optimized molecular structures of biscoumarins **1**–**4** with atomic numbering calculated at B3LYP level of theory are shown in Figure 3. For compound **4**, experimental geometric parameters and selected calculated geometric parameters under three different basis sets (6-31G*, 6-31+G**, and 6-311G*) are presented in Table 1. 

Under three different basis sets, the calculated results are very close with the average discrepancy of the selected bond lengths and bond angles between theoretical and experimental data is less than ± 0.02 Å and ± 2° respectively. B3LYP/6-31G* exhibited sufficient agreement with experimental data and lower computational cost, so further theoretical study was performed at this level.

**Table 1:** Experimental and calculated parameters of the selected bond lengths and bond angles of 3,3′-(3-fluoro-4-chlorobenzylidene)-bis-(4-hydroxycoumarin) (**4**).

	X-ray	6-31G*	6-31+G**	6-311G*
*R*(C_1_—O_1_)	1.224	1.234	1.236	1.226
*R*(C_1_—O_2_)	1.35	1.372	1.37	1.371
*R*(C_19_—O_4_)	1.221	1.23	1.233	1.223
*R*(C_19_—O_5_)	1.362	1.375	1.372	1.374
*R*(C_2_—C_10_)	1.508	1.526	1.526	1.526
*R*(C_10_—C_11_)	1.516	1.525	1.524	1.524
*R*(C_10_—C_20_)	1.536	1.539	1.539	1.537
*A*(C_1_—O_2_—C_9_)	121.11	121.67	121.7	121.7
*A*(O_1_—C_1_—O_2_)	116.05	115.7	115.9	115.95
*A*(C_18_—O_5_—C_19_)	121.34	121.9	121.89	121.92
*A*(O_4_—C_19_—O_5_)	114.64	115.67	115.85	115.96
*A*(C_2_—C_10_—C_20_)	114.84	115.8	115.94	115.88
*A*(C_2_—C_10_—C_11_)	113.67	112.59	112.77	112.68
*A*(C_11_—C_10_—C_20_)	113.79	114.51	114.61	114.57
*D*(C_1_—C_2_—C_10_—C_20_)	137.5	133.73	133.05	133.63
*D*(C_1_—C_2_—C_10_—C_11_)	89.01	91.83	92.01	91.65
*D*(C_12_—C_11_—C_10_—C_20_)	129.87	131.31	130.59	130.23
*D*(C_12_—C_11_—C_10_—C_2_)	96.14	93.64	93.85	94.44

#### 2.2.2. Estimation of the Single and Total HB Energies in Compounds **1**–**4**

We estimate single and total HB energies of compound 4 as an example. The global minimum structure is stabilized by two HBs (4ab); two higher energy structures is stabilized by one HB (4a and 4b) respectively.

From Table 2 we can see that, the O_6_—H_6_···O_1_ HB energy was estimated to be −51.402039 kJ/mol by the equation $\text{E}\left({\text{O}}_{6}—{\text{H}}_{6}\ldots {\text{O}}_{1}\right)={\text{E}}_{4\text{ab}}^{\text{coor}}-{\text{E}}_{4\text{a}}^{\text{coor}}$, is from the energy difference between 4ab and 4a (4a is a global minimum structure with O_3_—H_3_···O_4_ HB). Similarly, the O_3_—H_3_···O_4_ HB energy was calculated to be −65.4353365 kJ/mol by the equation $\text{E}\left({\text{O}}_{3}—{\text{H}}_{3}\ldots {\text{O}}_{4}\right)={\text{E}}_{4\text{ab}}^{\text{coor}}-{\text{E}}_{4\text{b}}^{\text{coor}}$, is from the energy difference between 4ab and 4b (4b was obtained from the global minimum structure 4ab, but H_3_ was rotated around the C_3_—O_3_ bond until O_3_—H_3_···O_4_ HB rupture occurred [12,13]).

**Table 2:** Total electronic energies (in hartree) and hydrogen bond (HB) energies (in kJ/mol) of hydrogen bonded conformers of compounds **1**–**4** calculated at B3LYP/6-31G* level of theory.

System	Total Electronic Energies ^a^	E(O_6_—H_6_···O_1_)	E(O_3_—H_3_···O_4_)	E(Total HB)
1ab	−1452.593097			−119.118935
1a	−1491.908094	−52.4391115		
1b	−1491.888121		−67.1051545	
2ab	−1603.031177			−122.1776425
2a	−1603.011081	−52.762048		
2b	−1603.004738		−69.4155945	
3ab	−4083.681309			−116.1757495
3a	−4083.66182	−51.1683695		
3b	−4083.656549		−65.00738	
4ab	−1972.171758			−116.8373755
4a	−1972.15218	−51.402039		
4b	−1972.146835		−65.4353365	

^a^ ZP corrected.

The total HB energy in compound **4**, calculated by the equation $\text{E}\left({\text{O}}_{3}—{\text{H}}_{3}\ldots {\text{O}}_{4}\right)+\text{E}\left({\text{O}}_{6}—{\text{H}}_{6}\ldots {\text{O}}_{1}\right)$, was estimated to be −116.8373755 kJ/mol. For compounds **1**–**3**, their total HB energies are −119.118935, −122.1776425 and −116.1757495 kJ·mol^−1^, respectively.

### 2.3. Minimal Inhibitory Concentration (MIC) Assay

For compounds **1**–**13**, one drug-sensitive *S. aureus* (*S. aureus* ATCC 29213) strain and three MRSA strains (MRSA XJ 75302, Mu50, USA 300 LAC), one drug-sensitive *S. epidermidis* strain (*S. epidermidis* ATCC 14990) and one methicillin-resistant *S. epidermidis* strain (MRSE XJ 75284) were used in the systematic analysis of their antibacterial activities *in vitro*. From Table 3 we can see that in all the synthesized compounds, biscoumarin derivatives **3** and **4** exerted more potent anti-bacterial activity against all tested strains expect for *E. Coli* with the MIC values of 8–16 μg/mL. Compared with compounds **1**–**13**, the MIC values of levofloxacin, ceftazidime, ceftriaxone, gentamicin, piperacillin and vancomycin against *S. aureus* (ATCC 29213) strain were lower (less than 8 μg/mL) but were higher against other strains at varying degrees.

**Table 3:** Minimum inhibitory concentrations (MIC) of compounds **1**–**13** and antibiotics in Mueller-Hinton broth culture.

Drugs	MIC (μg/mL)						
	*S. aureas*	MRSA	Mu50	LAC	*S. epidermidis*	MRSE	*E. Coli*
Compound **1**	>256	>256	>256	>256	>256	>256	>256
Compound **2**	>256	>256	>256	>256	>256	>256	>256
Compound **3**	16	16	8	8	8	8	>256
Compound **4**	8	8	8	8	8	8	>256
Compound **5**	>256	>256	>256	>256	>256	>256	>256
Compound **6**	>256	>256	>256	>256	>256	>256	>256
Compound **7**	>256	>256	>256	>256	>256	>256	>256
Compound **8**	>256	>256	>256	>256	>256	>256	>256
Compound **9**	>256	>256	>256	>256	>256	>256	>256
Compound **10**	>256	>256	>256	>256	>256	>256	>256
Compound **11**	>256	>256	>256	>256	>256	>256	>256
Compound **12**	>256	>256	>256	>256	>256	>256	>256
Compound **13**	>256	>256	>256	>256	>256	>256	>256
Levofloxacin	<0.125 (*S*)	4 (*R*)	4 (*R*)	8 (*R*)	0.125 (*S*)	0.125 (*S*)	0.25 (*S*)
Ceftazidime	8 (*S*)	>256 (*R*)	256 (*R*)	64 (*R*)	1 (*S*)	256 (*R*)	0.25 (*S*)
Ceftriaxone	2 (*S*)	>256 (*R*)	256 (*R*)	32 (*R*)	1 (*S*)	256 (*S*)	0.25 (*S*)
Gentamicin	0.12 (*S*)	64 (*R*)	32 (*R*)	0.25 (*S*)	0.25 (*S*)	32 (*R*)	2 (*S*)
Piperacillin	2 (*S*)	>128 (*R*)	>128 (*R*)	8 (*R*)	2 (*S*)	>128 (*R*)	2 (*S*)
Vancomycin	0.25 (*S*)	8 (*I*)	8 (*I*)	0.5 (*S*)	0.25 (*S*)	0.5 (*S*)	>256 (*R*)

*S* means drug susceptibility, *R* means drug resistance, *I* means intermediate resistance.

### 2.4. In Vitro Toxicity Measurement

**Figure 4:**
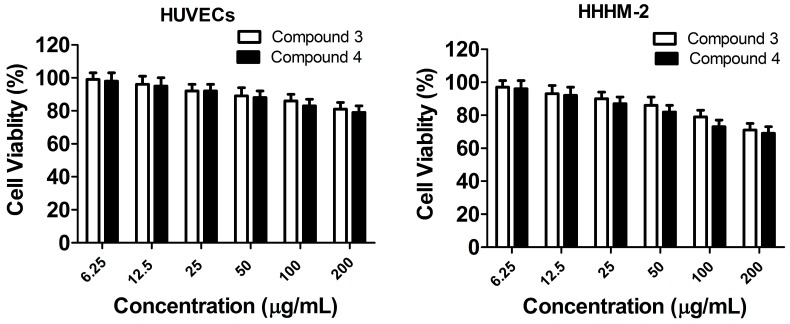
Cytotoxicity measurement of compounds **3** and **4** on the human umbilical vein endothelial cells (HUVECs) and human embryonic cardiomyocyte cell line CCC-HHM-2 (HHHM-2) *in vitro*.

To further explore the safety of the possible development, we investigated the cytotoxicity of compounds **3** and **4** to human umbilical vein endothelial cells (HUVECs) and human embryonic cardiomyocyte cell line CCC-HHM-2 (HHHM-2) in vitro. As shown in Figure 4, there was no obvious inhibitory effect of compounds 3 and 4 on cell viability at a concentration between 6.25 to 200 μg·mL^−1^, and the IC_50_ of compound **3** to the HUVECs and HHHM-2 was 1268.25 μg·mL^−1^ and 1036.56 μg·mL^−1^, the corresponding value of compound **4** was 1145.36 μg·mL^−1^ and 916.14 μg·mL^−1^. These results implied that compounds **3** and **4** had much less toxicity to mammalian cells, and had a relatively wide safety range for potential antimicrobial applications.

### 2.5. Discussion

Methicillin-resistant *S. aureus* (MRSA) and is the cause of major outbreaks and epidemics in hospitalized patients because of the emergence, spread, and rapid evolution of resistance genes developing among pathogens. As such, the mortality and morbidity rates of patients affected by MRSA are high. However, the emergence of vancomycin-resistant *S. aureus* and treatment failure of MRSA infections urgently requires developing new antimicrobials.

In this work, among two kinds of compounds, novel biscoumarin derivatives **3** and **4** were demonstrated to be capable of remarkably inhibiting the growth of all tested strains expect for *E. coli* with the MIC value of 8–16 μg/mL. However, compared with compounds **3** and **4**, other biscoumarins **1** and **2** and dihydropyran derivatives **5**–**13** almost have no antibacterial activities. In addition, the results of *in vitro* toxicity study of compounds **3** and **4** on HUVECs and HHHM-2 revealed that the two compounds had a relatively wide safety range for potential antimicrobial applications with much less toxicity to mammalian cells.

X-ray structural analysis also showed that biscoumarins **1**–**4** were stabilized by two asymmetrical intramolecular O—H···O HBs, which were considered as important factors in assisting the molecule to attain the correct configuration for biological activity [14]. The total HB stabilization energies in compounds **1**–**4** were estimated to be −119.118935, −122.1776425, −116.1757495 and −116.8373755 kJ·mol^−1^, respectively. These values suggest that the most potent antibacterial activity in compounds **3** and **4** was consistent with their weakest HB strengths.

## 3. Experimental Section

### 3.1. Apparatus and Materials

IR spectra (400–4000 cm^−1^) were obtained using a Brucker Equinox-55 spectrophotometer (Fremont, CA, USA). ^1^H-NMR spectra were obtained using a Varian Inova-400 spectrometer (at 400 MHz) (Palo Alto, CA, USA). Mass spectra were obtained using a micrOTOF-Q II mass spectrometer (Bruker). The melting points were taken on a XT-4 micro melting apparatus (Taike Company, Beijing, China), and the thermometer was uncorrected.

All antibiotics used were purchased from the National Institute for the Control of Pharmaceutical and Biological Products (Beijing, China). All other chemicals and solvents were of analytical grade.

MRSA (XJ 75302) and MRSE (XJ 75284) were isolated from cultures of sputum samples from patients in Xijing Hospital (Xi’an, China). *S. aureus* (ATCC 29213), *S. epidermidis* (ATCC 14990) and *E. coli* (ATCC 25922) strains were purchased from the Chinese National Center for Surveillance of Antimicrobial Resistance. Mu50 (ATCC 700699) and USA 300 (LAC) were purchased from MicroBiologics (St. Cloud, MN, USA).

### 3.2. Synthesis and Characterization of Compounds **1**–**13**

Biscoumarins **1**–**4** were synthesized according to the methods of a previous report [15]. A mixture of 3,4-dimethylbenzaldehyde (3-hydroxy-4-methoxybenzaldehyde, 3-bromo-4-fluorobenzaldehyde, 3-fluoro-4-chlorobenzaldehyde) (10 mmol) and 4-hydroxycoumarin (20 mmol) was dissolved in 100 mL of EtOH. A few drops of piperidine were added, and the mixture was stirred for 3 h at room temperature. After reaction completion as determined by TLC, water was added until precipitation occurred. The solid was filtered off and then recrystallized from ethanol to give compounds **1**–**4**.

*3,3′-(3,4-Dimethylbenzylidene)-bis-(4-hydroxycoumarin)* (**1**): m.p. 227–228 °C. IR (KBr pellet cm^−1^): 2922, 1671, 1601, 1561, 1342, 1303, 1094, 766 cm^−1^. ^1^H-NMR (CDCl_3_, δ, ppm): 11.507 (s, 1H), 11.268 (s, 1H), 8.015–8.065 (q, 2H), 7.606–7.647 (t, 2H), 7.404–7.425 (d, 4H), 7.067–7.088 (d, 1H), 6.950 (s, 2H), 6.049 (s, 1H), 2.204 (s, 3H), 2.241 (s, 3H). HRMS (ESI^+^): *m*/*z*: calcd for C_27_H_20_O_6_: 463.1152 [M + Na]^+^; found: 463.1135.

*3,3′-(3-Hydroxy-4-methoxybenzylidene)-bis-(4-hydroxycoumarin)* (**2**): m.p. 246–247 °C. IR (KBr pellet cm^−1^): 3530, 2608, 1667, 1615, 1567, 1512, 1453, 1350, 1269, 1215, 1095, 1028, 763 cm^−1^. ^1^H-NMR (CDCl_3_, δ, ppm): 11.612 (s, 1H), 11.277 (s, 1H), 8.034–8.070 (d, 2H), 7.623–7.666 (m, 2H), 7.414–7.435 (d, 4H), 6.799–6.819 (t, 2H), 6.708–6.729 (t, 1H), 6.047 (s, 1H), 5.612 (s, 1H), 3.895 (s, 3H). HRMS (ESI^+^): *m*/*z*: calcd for C_26_H_18_O_8_: 481.0894 [M + Na]^+^; found: 481.7855.

*3,3′-(3-Bromo-4-fluorobenzylidene)-bis-(4-hydroxycoumarin)* (**3**): m.p. 215–216 °C. IR (KBr pellet cm^−^^1^): 1666, 1617, 1567, 1495, 1348, 1097, 1047, 763 cm^−1^. ^1^H-NMR (DMSO-*d*_6_, δ, ppm): 7.885–7.901 (d, 3H), 7.763 (s, 2H), 7.562–7.601 (t, 2H), 7.279–7.357 (m, 4H), 6.403 (s, 1H). HRMS (ESI^+^): *m*/*z*: calcd for C_25_H_14_BrFO_6_: 530.9850 [M + Na]^+^; found: 530.9865.

*3,3′-(3-Fluoro-4-chlorobenzylidene)-bis-(4-hydroxycoumarin)* (**4**): m.p. 259–260 °C. IR (KBr pellet cm^−1^): 3448, 3083, 1790, 1670, 1611, 1561, 1489, 1413, 1259, 1172, 1064, 863, 767 cm^−1^. ^1^H-NMR (CDCl_3_, δ, ppm): 11.626 (s, 1H), 11.344 (s, 1H), 8.020–8.107 (m, 2H), 7.656–7.699 (m, 2H), 7.340–7.458 (m, 5H), 6.972–7.055 (m, 2H), 6.054 (s, 1H). HRMS (ESI^+^): *m*/*z*: calcd for C_25_H_14_ClFO_6_: 487.0355 [M + Na]^+^; found: 487.0433.

Dihydropyran derivatives (**5**–**13**) were also synthesized according to a reported procedure [16]. A mixture of 4-hydroxycoumarin (1-naphthalenol, 1,1-dimethyl-3,5-cyclohexanedione) (10 mmol), aromatic aldehydes (10 mmol), malononitrile (10 mmol) and 4-(dimethylamino)pyridine (DMAP) (1 mmol) in ethanol (100 mL) was refluxed for 2–3 h and then cooled to room temperature. The solid was filtered off and then recrystallized from ethanol to give compounds **5**–**13**. 

*2-Amino-4-(4-hydroxyphenyl)-3-cyano-5-oxo-4H,5H-pyrano[3,2c]chromene* (**5**): m.p. 269–270 °C. IR (KBr pellet cm^−1^): 3511, 3279, 2191, 1694, 1670, 1606, 1382, 1262, 1165, 1069, 757 cm^−1^. ^1^H-NMR (DMSO-*d*_6_, δ, ppm): 9.387 (s, 1H), 7.879–7.902 (q, 1H), 7.684–7.727 (m, 1H), 7.447–7.507 (q, 2H), 7.356 (s, 2H), 7.034–7.055 (d, 2H), 6.679–6.700 (d, 2H), 4.331 (s, 1H). HRMS (ESI^+^): *m*/*z*: calcd for C_19_H_12_N_2_O_4_: 355.0689 [M + Na]^+^; found: 355.0680.

*2-Amino-4-(4-chlorophenyl)-3-cyano-5-oxo-4H,5H-pyrano[3,2c]chromene* (**6**): m.p. 254–255 °C. IR (KBr pellet cm^−1^): 3386, 3312, 3190, 2193, 1714, 1675, 1610, 1490, 1377, 1060, 759 cm^−1^. ^1^H-NMR (DMSO-*d*_6_, δ, ppm): 7.889–7.913 (q, 1H), 7.703–7.746 (m, 1H), 7.461–7.521 (m, 4H), 7.359–7.386 (m, 2H), 7.301–7.328 (m, 2H), 4.493 (s, 1H). HRMS (ESI^+^): *m*/*z*: calcd for C_19_H_11_ClN_2_O_3_: 373.0350 [M + Na]^+^; found: 373.2438. 

*2-Amino-4-(4-nitrophenyl)-3-cyano-5-oxo-4H,5H-pyrano[3,2c]chromene* (**7**): m.p. 259–260 °C. IR (KBr pellet cm^−1^): 3688, 2191, 1718, 1662, 1509, 1341, 1253, 1053, 957, 901, 765 cm^−1^. ^1^H-NMR (DMSO-*d*_6_, δ, ppm): 8.175–8.196 (d, 2H), 7.910–7.933 (q, 1H), 7.723–7.766 (m, 1H), 7.475–7.611 (m, 6H), 4.682 (s, 1H). HRMS (ESI^+^): *m*/*z*: calcd for C_19_H_11_N_3_O_5_: 384.0591 [M + Na]^+^; found: 384.0533. 

*2-Amino-4-(3-phenoxyphenyl)-4H-benzo[h]chromene-3-carbonitrile* (**8**): m.p. 173–174 °C. IR (KBr pellet cm^−1^): 3337, 1627, 1573, 1488, 1445, 1378, 1240, 1189, 1105, 1025, 892, 758 cm^−1^. ^1^H-NMR (DMSO-*d*_6_, δ, ppm): 8.221–8.241 (d, 1H), 7.899–7.919 (d, 1H), 7.567–7.661 (m, 3H), 7.301–7.389 (m, 3H), 7.112–7.206 (m, 4H), 6.979–7.008 (t, 4H), 6.796–6.822 (q, 1H), 4.938 (s, 1H). HRMS (ESI^+^): *m*/*z*: calcd for C_26_H_18_N_2_O_2_: 413.1260 [M + Na]^+^; found: 413.1255.

*2-Amino-4-(4-methanesulfonylphenyl)-4H-benzo[h]chromene-3-carbonitrile* (**9**): m.p. 244–245 °C. IR (KBr pellet cm^−1^): 3001, 1629, 1573, 1487, 1412, 1304, 1234, 1190, 1150, 1103, 1016, 764 cm^−1^. ^1^H-NMR (DMSO-*d*_6_, δ, ppm): 8.246–8.267 (d, 1H), 7.887–7.925 (t, 3H), 7.602–7.683 (m, 3H), 7.523–7.544 (d, 2H), 7.039 (s, 2H), 7.116–7.138 (d, 1H), 5.106 (s, 1H), 3.203 (s, 3H). HRMS (ESI^+^): *m*/*z*: calcd for C_21_H_16_N_2_O_3_S: 399.0774 [M + Na]^+^; found: 399.0723.

*2-Amino-4-(4-nitrophenyl)-4H-benzo[h]chromene-3-carbonitrile* (**10**): m.p. 241–242 °C. IR (KBr pellet cm^−1^): 3477, 1651, 1512, 1404, 1350, 1185, 1101, 1022, 801, 770 cm^−1^. ^1^H-NMR (DMSO-*d*_6_, δ, ppm): 8.198–8.272 (q, 3H), 7.905–7.926 (d, 1H), 7.538–7.685 (m, 5H), 7.343 (s, 2H), 7.106–7.128 (d, 1H), 5.164 (s, 1H). HRMS (ESI^+^): *m*/*z*: calcd for C_20_H_13_N_3_O_3_: 366.0849 [M + Na]^+^; found: 366.0831. 

*2-Amino-4-(4-methoxyphenyl)-3-cyano-7,7-dimethyl-5-oxo-4H-5,6,7,8-tetrahydrobenzo[b]pyran* (**11**): m.p. 209–210 °C. IR (KBr pellet cm^−1^): 3320, 3187, 2198, 1685, 1649, 1606, 1582, 1509, 1462, 1411, 1370, 1247, 1033, 842 cm^−1^. ^1^H-NMR (DMSO-*d_6_*, δ, ppm): 7.045–7.067 (d, 2H), 6.954 (s, 2H), 6.834–6.855 (d, 2H), 4.124 (s, 1H), 3.718 (s, 3H), 2.452–2.508 (q, 2H), 2.229–2.269 (d, 1H), 2.075–2.115 (d, 1H), 1.039 (s, 3H), 0.952 (s, 3H). HRMS (ESI^+^): *m*/*z*: calcd for C_19_H_20_N_2_O_3_: 347.1366 [M + Na]^+^; found: 347.1324. 

*2-Amino-3-cyano-4-phenyl-7,7-dimethyl-5-oxo-4H-5,6,7,8-tetrahydrobenzo[b]pyran* (**12**): m.p. 219–220 °C. IR (KBr pellet cm^−1^): 3213, 2961, 2199, 1689, 1603, 1492, 1454, 1413, 1372, 1217, 1035, 838 cm^−1^. ^1^H-NMR (DMSO-*d*_6_, δ, ppm): 7.273–7.311 (t, 2H), 7.136–7.205 (m, 3H), 6.999 (s, 2H), 4.176 (s, 1H), 2.503–2.526 (m, 1H), 2.240–2.281 (d, 1H), 2.130 (s, 1H), 2.092 (s, 1H), 1.046 (s, 3H), 0.963 (s, 3H). HRMS (ESI^+^): *m*/*z*: calcd for C_18_H_18_N_2_O_2_: 317.1260 [M + Na]^+^; found: 317.1344. 

*2-Amino-4-(2,4-dinitrophenyl)-3-cyano-7,7-dimethyl-5-oxo-4H-5,6,7,8-tetrahydrobenzo[b]pyran* (**13**): m.p. 217–218 °C. IR (KBr pellet cm^−1^): 3422, 2202, 1682, 1664, 1602, 1537, 1361, 1216, 1044 cm^−1^. ^1^H-NMR (DMSO-*d*_6_, δ, ppm): 8.619–8.625 (d, 1H), 8.434–8.462 (m, 1H), 7.707–7.729 (d, 1H), 7.374 (s, 2H), 4.989 (s, 1H), 2.202–2.243 (d, 1H), 2.091 (s, 2H), 2.009–2.050 (d, 1H), 1.027 (s, 3H), 0.898 (s, 3H). HRMS (ESI^+^): *m*/*z*: calcd for C_18_H_16_N_4_O_6_: 407.0962 [M + Na^+^]; found: 407.0945.

### 3.3. X-ray Crystallography

For X-ray diffraction experiments, single crystals of compounds **4**, **5**, **8** and **12** were both grown from methanol. The X-ray diffraction data were collected on a Bruker SMART APEX II CCD diffractometer equipped with a graphite monochromated Mo Kα radiation (λ = 0.71073 Å) by using the *ω*-2*θ* scan technique at room temperature. The structure was solved by direct methods using SHELXL-97 [17] and refined using the full-matrix least squares method on *F*^2^ with anisotropic thermal parameters for all non-hydrogen atoms by using SHELXL-97. Hydrogen atoms were generated geometrically. The crystal data and details concerning data collection and structure refinement are given in Table 4. Molecular illustrations were prepared using the XP package. Parameters in CIF format are available as Electronic Supplementary Publication from Cambridge Crystallographic Data Centre.

**Table 4:** Crystal data, data collection and structure refinement of compounds **4**, **5**, **8** and **12**.

	Compound 4	Compound 5	Compound 8	Compound 12
Formula	C_25_H_14_ClFO_6_	C_19_H_12_N_2_O_4_	C_26_H_18_N_2_O_2_	C_18_H_18_N_2_O_2_
*M*r	464.0463	332.0797	390.1368	294.1368
Crystal system	Monoclinic	Monoclinic	Monoclinic	Monoclinic
Space group	P2_1_/c	P2_1_/c	P2_1_/c	P2_1_/c
*a*/Å	10.1874(7)	8.8632(6)	11.908(3)	11.2938(5)
*b*/Å	10.1667(6)	13.1743(8)	15.4731(17)	9.4591(5)
*c*/Å	20.0790(12)	13.0565(6)	11.9343(18)	14.9132(7)
α/°	90	90	90	90
β/°	96.571(7)	94.484(6)	116.15(2)	99.304(4)
γ/°	90	90	90	90
*V*/Å^3^	2066.0(2)	1519.89(15)	1973.9(6)	1572.21(13)
*Z*	4	4	4	4
*D*_calc_/g·cm^−3^	1.491	1.452	1.314	1.244
μ(Mo Kα)/mm^−1^	0.236	0.104	0.084	0.082
θ range/°	2.70 to 25.00	2.78 to 25.00	2.63 to 25.00	2.56 to 25.00
Reflections collected	7346	5980	4969	6337
No. unique data [*R*(int)]	3621 [0.0256]	2670 [0.0263]	3099 [0.0186]	2775 [0.0280]
No. data with *I* ≥ 2*σ*(*I*)	2293	1991	2029	2109
*R*_1_	0.0501	0.0453	0.0542	0.0465
*ωR*_2_(all data)	0.1704	0.1559	0.1561	0.1627
CCDC	1032644	1032645	1032646	1032647

### 3.4. Quantum Chemical Calculations

All calculations were carried out using the Gaussian 09 package [18]. Density functional theory (DFT) [19], Becke’s three-parameter hybrid function (B3LYP) [20], and LYP correlation function [21,22] were used to fully optimize all the geometries on the energy surface without constraints. To obtain precise results that are in conjunction with experimental results, three basis sets, namely 6-31G*, 6-31+G**, and 6-311G*, were tested. Frequency calculations at the B3LYP (with basis sets 6-31G*) level of theory were carried out to confirm stationary points as minima and to obtain the zero-point energies and the thermal correlation data at 1 atm and 298 K.

### 3.5. Minimal Inhibitory Concentration (MIC) Assay

Based on the CLSI broth microdilution method [23], the determination of minimum inhibitory concentrations (MICs) via microdilution assay was performed in sterilized 96-well polypropylene microtiter plates (Sigma-Aldrich, St. Louis, MO, USA) in a final volume of 200 μL. Bacteria were grown overnight in nutrient broth. Mueller-Hinton (MH) broth (100 μL) containing bacteria (5 × 10^5^ CFU/mL) was added to 100 μL of the culture medium containing the test compound (0.12 μg/mL to 256 μg/mL in serial twofold dilutions). The plates were incubated at 37 °C for 20 h in an incubator. About 50 µL of 0.2% triphenyl tetrazolium chloride (TTC), a colorimetric indicator, was added to each well of microtiter plates and incubated at 35 °C for 1.5 h. The TTC-based MIC was determined as the lowest concentration of oxacillin that showed no red color change indicating complete growth inhibition. The data were from three repeated experiments.

### 3.6. Cytotoxicity Assay

The cytotoxicity of compounds **3** and **4** to the human umbilical vein endothelial cells (HUVECs) and the human embryonic cardiomyocyte cell line CCC-HHM-2 (HHHM-2) was determined by 3-(4,5-dimethyl-2-thiazolyl)-2,5-diphenyl-2-H-tetrazolium bromide (MTT) staining. Briefly, cells (5 × 10^3^ cells per well) were seeded in a 96-well plate with 100 μL DMEM medium with 20% fetal bovine serum (FBS) in each well for 12 h and then treated with or without compounds at various concentrations (12.5–800 μg·mL^−^^1^) for 24 h. After treatment, MTT solution (final concentration, 0.5%) was added and cells were incubated for another 4 h at 37 °C. 150 μL DMSO was added to each well after removal of the supernatant and the absorbance at 490 nm was measured with a microplate reader.

## 4. Conclusions

With the emergence of methicillin-resistant, vancomycin intermediate-resistant, or multi-drug-resistant *S. aureus*, more appropriate antibiotics need to be developed. Our results showed that biscoumarin derivatives **3** and **4** have better anti-bacterial efficiency with minimum MIC value of 8–16 μg/mL in all synthesized compounds, which is may be that there are two intramolecular O—H···O HBs in their structures and two strong electron-withdrawing atoms (Br and F; F and Cl) further weakens the total HB energy.
